# Fumigaclavine C ameliorates liver steatosis by attenuating hepatic de novo* lipogenesis* via modulation of the RhoA/ROCK signaling pathway

**DOI:** 10.1186/s12906-023-04110-9

**Published:** 2023-08-16

**Authors:** Wanguo Yu, Yaxin Gao, Zaoya Zhao, Xiufeng Long, Yi Yi, Shuo Ai

**Affiliations:** 1 Key Laboratory for Processing of Sugar Resources of Guangxi Higher Education Institutes, Guangxi University of Science and Technology, 268 Donghuan Road, Liuzhou, 545006 People’s Republic of China; 2 Guangxi Key Laboratory of Green Processing of Sugar Resources, Guangxi University of Science and Technology, 268 Donghuan Road, Liuzhou, 545006 People’s Republic of China

**Keywords:** Fumigaclavine C, Non-alcoholic fatty liver disease, Anti-obesity, Protein prenylation

## Abstract

**Background:**

Non-alcoholic fatty liver disease (NAFLD) has been well defined as a common chronic liver metabolism disorder. Statins as a first-line therapeutic treatment had some side effects. Here, we found that Fumigaclavine C (FC) was collected from endophytic *Aspergillus terreus* via the root of *Rhizophora stylosa* (Rhizophoraceae), had potential anti-adipogenic and hepatoprotective effects both in vitro and in vivo without obvious adverse side effects. However, the mechanisms of the prevention and management of FC for hepatic steatosis are incompletely delineated.

**Methods:**

The pharmacodynamic effects of FC were measured in high-fat diet (HFD)-induced obese mice. Liver index and blood biochemical were examined. Histopathological examination in the liver was performed by hematoxylin & eosin or oil red O. The levels of serum TG, TC, LDL-c, HDL-c, FFA, T-bili, ALT, AST, creatinine, and creatine kinase were estimated via diagnostic assay kits. The levels of hepatic lipid metabolism-related genes were detected via qRT-PCR. The expression levels of hepatic de novo* lipogenesis* were quantitated with Western blot analysis.

**Results:**

FC-treatment markedly reduced hepatic lipid accumulation in HFD-induced obese mice. FC significantly attenuated the hepatic lipid metabolism and ameliorated liver injury without obvious adverse side effects. Moreover, FC also could dose-dependently modulate the expressions of lipid metabolism-related transcription genes. Mechanically, FC notably suppressed sterol response element binding protein-1c mediated de novo* lipogenesis* via interfering with the RhoA/ROCK signaling pathway by decreasing the levels of geranylgeranyl diphosphate and farnesyl diphosphate.

**Conclusions:**

These findings suggested that FC could improve hepatic steatosis through inhibiting de novo* lipogenesis* via modulating the RhoA/ROCK signaling pathway.

**Supplementary Information:**

The online version contains supplementary material available at 10.1186/s12906-023-04110-9.

## Background

Non-alcoholic fatty liver disease (NAFLD) has been well defined as a chronic liver metabolism disorder and its prevalence is a serious and increasing clinical problem worldwide, closely related to obesity, diabetes, hyperlipidemia, and hypertension [1]. It contributes to various liver pathologies which range from nonalcoholic simple steatosis, nonalcoholic steatohepatitis (NASH), cirrhosis, to liver cancer [2]. Of these types, simple hepatic steatosis status could be reversed through effective treatment and prevented it from progressing to some more severe stages [3]. Hence, it is an allimportant step in the prevention of hepatic steatosis via ameliorating lipid accumulation and blocking the hepatic lipid metabolism-related genes expressions [4].

Until now, various alternative NAFLD therapeutic strategies including lifestyle intervention and pharmaceutical treatment are either insufficient or have side effects [5, 6]. Although lifestyle intervention is an impactful way to ameliorate NAFLD, prolonged prevention works in half [7, 8]. Currently, some candidates containing natural products and chemical compounds are used for clinical treatment of NAFLD. For instance, statins, HMG-CoA reductase inhibitor, had been used for management of NAFLD-associated hypercholesterolemia [9, 10]. Nevertheless, several safety concerns have been reported for statins. Long-term statins treatment had side effects including liver injury and myopathy [11, 12]. Several articles have reported that statins deplete cholesterol, geranylgeranyl diphosphate (GGPP), and farnesyl diphosphate (FPP) at the beginning of mevalonate pathway [13, 14]. GGPP and FPP are used for the prenylation of proteins at their carboxyl-terminal CAAX motif, like RhoA and Ras [15]. But, various issues remain unresolved elucidating the precise mechanisms of treatment chemical drugs for NAFLD [16, 17]. To overcome above issues, emerging researches have focused on natural products as a selectable effective therapeutic strategy without significant adverse side effects [18, 19].

**Fig. 1:**
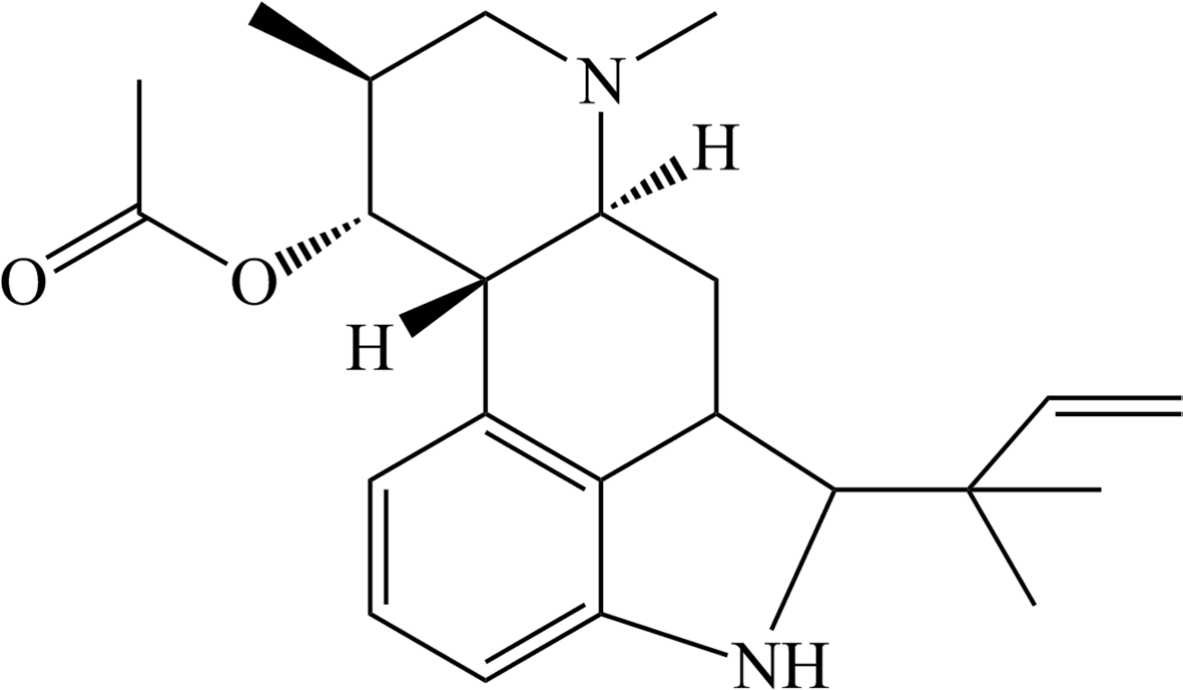
The chemical structure of fumigaclavine C

Teas have long been considered as a dietary supplementation [20]. *Rhizophora stylosa* (Rhizophoraceae) has been considered as a common edible plant whose roots and leaves were used as an ingredient for Chinese herbal tea [21]. As shown in Fig. 1, fumigaclavine C (FC) which is an indole alkaloid, is collected from endophytic *Aspergillus terreus* (strain No. FC118) via the root of *Rhizophora stylosa* (Rhizophoraceae). It has various health beneficial effects, including anti-tumor [22], anti-atherosclerosis [23], anti-inflammation [24–27], anti-adipogenic [28], immunosuppressive activity [29], and hepatoprotective activity [30]. Our previous paper indicated that FC had potential anti-adipogenic effect in obese animal model [28]. However, the precise molecular mechanisms of the adipogenesis and lipolysis of FC are incompletely delineated. Herein, the aim of this study was to elucidate the effectivity of FC in the prevention and management of NAFLD both in vitro and in vivo without obvious adverse side effects.

## Methods

### Materials

FC (≥ 99.5%) was collected from endophytic *Aspergillus terreus* (strain No. FC118) via the root of *Rhizophora stylosa* (Rhizophoraceae). The anti-RhoA, anti-prenyl, anti-ROCK, anti-sterol response element binding protein-1c (Srebp-1c), anti-α-tubulin antibodies, and secondary antibody were purchased from Cell Signaling Technology (Danvers, MA, USA). Oleic acid, palmitic acid, and simvastatin (Sim) was purchased from Sigma Aldrich Company (St. Louis, MO, USA). Total cholesterol (TC), Triglyceride (TG), low density lipoprotein-cholesterol (LDL-c), high density lipoprotein-cholesterol (HDL-c), free fatty acid (FFA), total bilirubin (T-bili), alanine transaminase (ALT), aspartate transaminase (AST), creatinine, creatine kinase, and Cell Counting Kit-8 (CCK-8) were measured by diagnostic assay kits from Nanjing Jiancheng Company (NJ, China).

### Experimental animals design

**Fig. 2:**
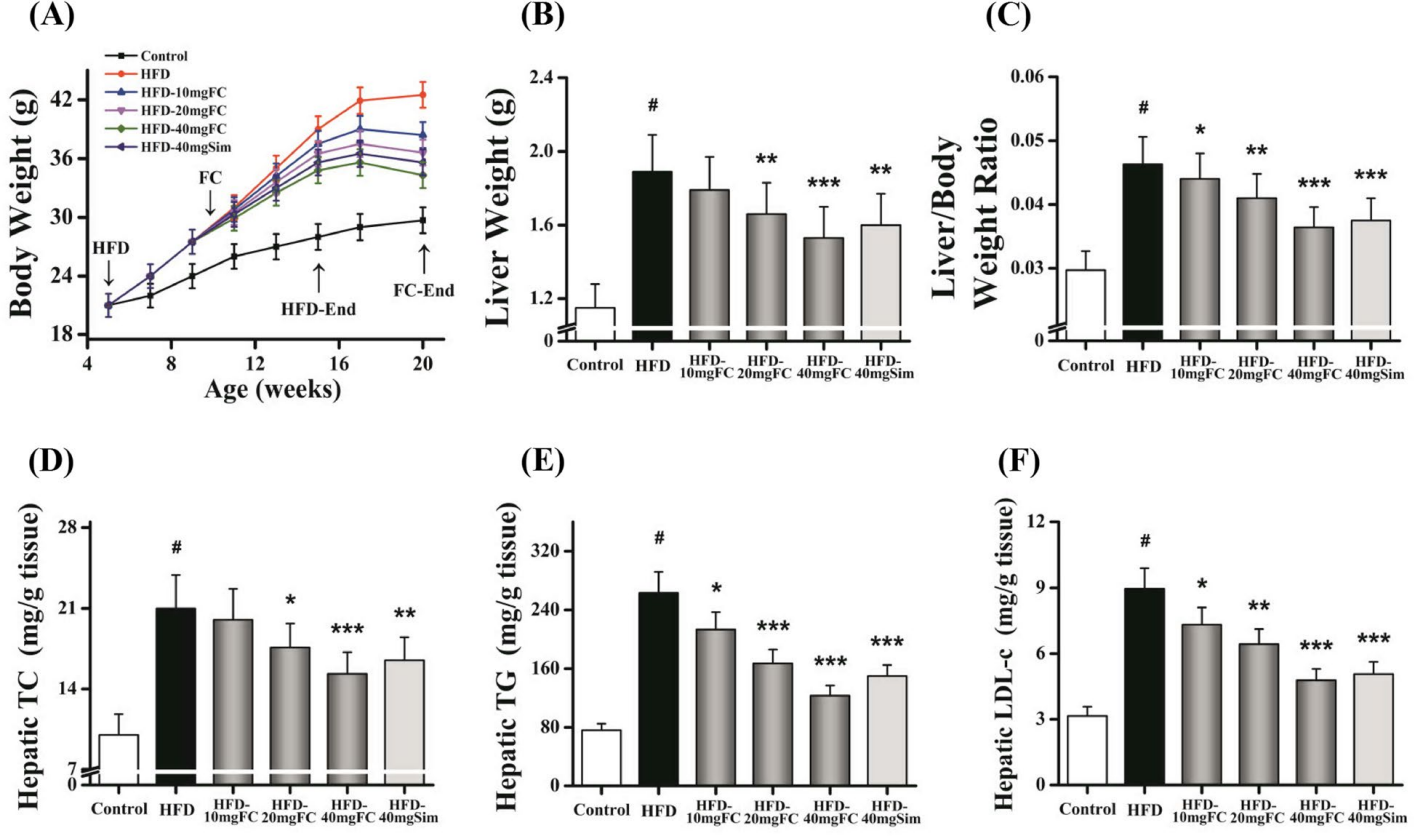
FC improves hepatic steatosis in high-fat diet (HFD)-induced obese mice. **A** The time course of body weight within HFD-induced and FC administration. **B** The liver weight was examined. **C** The liver/body weight ratio was calculated. **D**-**F** The levels of hepatic TC, TG, and LDL-c were detected. TC, total cholesterol; TG, Triglyceride; LDL-c, low density lipoprotein-cholesterol. Simvastatin (Sim, 40 mg/kg) acted as the positive control group. Each value represents as means ± S.E.M. of triplicate experiments. ^#^ *P* < 0.01 as compared with the control group. ^*^ *P* < 0.05, ^**^ *P* < 0.01, and ^***^ *P* < 0.001 as compared with the HFD-induced group

All of male C57BL/6 mice were purchased from SJA Laboratory Animal Co., Ltd. (Hunan, China). The four-week-old mice were acclimatized to the experimental facility for one week. The mice were either fed with regular chow (RC, 3.6 kcal/g, 10% fat, 14% protein, and 76% carbohydrates) or high fat diet (HFD, 5.5 kcal/g, 50% fat, 14% protein, and 36% carbohydrates [Shanghai FBSH Biological Pharmaceutical Co., Ltd., Shanghai, China]) for ten weeks to induce NAFLD steatosis model. Experiments with animals were performed following the animal ethics guidelines of the Institutional Animal Ethics Committee. The mice were equally and randomly divided into six groups (ten mice / group): control group (only DMSO), HFD-induced group (only DMSO), HFD-10 mg FC (10 mg/kg of body weight), HFD-20 mg FC, HFD-40 mg FC, and HFD-40 mg Sim. Sim and FC were dissolved into DMSO (final concentration ≤ 5%). FC was administrated via intraperitoneal injection. The injection was performed three times per week for ten weeks. Experimental animals procedures were approved as follow Fig. 2 A [31]. All experimental procedures were approved by the Institutional Animal Care and Use Committee of Liuzhou General Hospital (Protocol No. 1019–3).

### Cell culture and stimulation

AML-12 cells were purchased from American Type Culture Collection (ATCC, Rockville, MD, USA). The cells were cultured in DMEM/F12 medium containing fetal bovine saline (FBS, 10%), penicillin (100 U/mL), and streptomycin (100 μm/mL) at 37 °C with 5% CO_2_ [32]. Cell viability was evaluated via CCK-8 kit following the manufactures’ instructions. Hepatocytes were stimulated with 500 mM free fatty acids (FFA, oleic acid: palmitic acid = 2:1) for 48 h. Sim and FC were dissolved into DMSO (final concentration ≤ 0.1%). FC was administered with indicated concentrations for 24 h.

### Biochemical data measurement

Body weight data were collected twice a week. Hepatocytes/hepatic TC, TG and LDL-c were respectively examined via assay kits following the manufactures’ instructions. The blood samples were collected from orbital venous plexus and centrifuged at 4000 rpm for 15 min at 4 °C. The levels of serum TG, TC, LDL-c, HDL-c, FFA, T-bili, ALT, AST, creatinine, and creatine kinase were respectively measured via diagnostic assay kits following the manufactures’ instructions.

### Histopathological analysis

Liver tissues were fixed in 10% formalin saline, embedded in paraffin, and processed following routine histology procedures. Tissue samples (6–7 μm) were stained with hematoxylin & eosin (H & E) or oil red O according to standard protocol by histology, and observed via light microscope (Nikon, Tokyo, Japan).

### Quantitative real-time polymerase chain reaction (qRT-PCR)

Total RNA was extracted with TRIzol reagent and reversed transcribed with Prime ScriptTM. RT reagent following the manufactures’ protocol (Invitrogen, Carlsbad, CA, USA). Quantitative real-time PCR was conducted using the Quanti Fast SYBR Green PCR Kit (Qiagen, Valencia, CA, USA). The list of PCR primers was shown in Table 1. All data were normalized by β-actin and performed in triplicate.

**Table 1:** Sequences of primers used for the real-time PCR analysis

Gene	Primers	Sequence
Srebp-1c	Forward	5′-CTGTTGGTGCTCGTCTCCT-3′
	Reverse	5′-TTGCGATGCCTCCAGAAGTA-3′
Ppar-γ	Forward	5′-GATGACAGCGACTTGGCAAT-3′
	Reverse	5′-TGTAGCAGGTTGTCTTGAATGT-3′
Fas	Forward	5′-ATCCTGGCTGACGAAGACTC-3′
	Reverse	5′-TGCTGCTGAGGTTGGAGAG-3′
Acc	Forward	5′-GATGAACCATCTCCGTTGGC-3′
	Reverse	5′-CCCAATTATGAATCGGGAGTGC-3′
Cpt1	Forward	5′-CCAGGCTACAGTGGGACATT-3′
	Reverse	5′-GAACTTGCCCATGTCCTTGT-3′
Ppar-α	Forward	5′-GGAGCGTTGTCTGGAGGTT-3′
	Reverse	5′-GAAGTGGTGGCTAAGTTGTTGA-3′
Mcad	Forward	5′-AGGTTTCAAGATCGCAATGG-3′
	Reverse	5′-CTCCTTGGTGCTCCACTAGC-3′
CD36	Forward	5′-ATGGGCTGTGATCGGAACTG-3′
	Reverse	5′-GTCTTCCCAATAAGCATGTCTCC-3′
Fabp1	Forward	5′-ATGAACTTCTCCGGCAAGTACC-3′
	Reverse	5′-CTGACACCCCCTTGATGTCC-3′
Fatp1	Forward	5′-CACGATCCCGTGCATCTTCC-3′
	Reverse	5′-AGCATTGGAGTAGGTGTCCAG-3′
ApoB	Forward	5′-TTGGCAAACTGCATAGCATCC-3′
	Reverse	5′-TCAAATTGGGACTCTCCTTTAGC-3′
ApoE	Forward	5′-CTCCCAAGTCACACAAGAACTG-3′
	Reverse	5′-CCAGCTCCTTTTTGTAAGCCTTT-3′
Mttp	Forward	5′-CTCTTGGCAGTGCTTTTTCTCT-3′
	Reverse	5′-GAGCTTGTATAGCCGCTCATT-3′
β-actin	Forward	5′-TGCTGTCCCTGTATGCCTCT-3′
	Reverse	5′-TGATGTCACGCACGATTTCC-3′

### Western blot analysis

Hepatocytes/hepatic protein extracts were respectively collected according to the manufactures’ protocol (Protein Extraction Kit, Pierce Biotechnology Inc., Nepean, Canada). Protein samples were quantified via using an Enhanced Bicinchoninic Acid Protein Assay Kit (Beyotime, Jiangsu, China). The harvested protein extracts were separated by SDS-PAGE and transferred to polyvinylidene difluoride (PVDF) membranes (Millipore, Bedford, MA). The PVDF membranes were blocked with 5% non-fat milk and incubated with anti-RhoA, anti-prenyl, anti-ROCK, anti-Srebp-1c, and anti-α-tubulin antibodies at 4°C overnight. The PVDF membranes were washed and exposed to secondary antibodies [33]. Finally, the blots were normalized via β-actin and quantitated with LI-COR Odyssey Analysis software.

### Protein prenylation examination

The levels of GGPP and FPP were examined according to previously reported protocol by HPLC–MS/MS in AML-12 cell [34]. To evaluate RhoA prenylation, hepatocyte subcellular fractionation was collected following a Triton X-114 partition method [35]. Briefly, the cells were directly lysed in 2% Triton X-114 on ice for 30 min. To fractionate the lipid-rich cell membrane, 500 μg protein samples were partitioned with 0.1% Triton X-114 at 37°C for 10 min. Subcellular phases were immunoprecipitated with anti-RhoA and anti-prenyl, then with protein A/G coupled to agarose beads. After a thorough wash, cell samples were measured to immunoblotting analysis.

### Statistical analysis

Results were represented as means ± S.E.M. or a percentage of triplicate tests. All data were performed via one-way ANOVA followed by the Newman-Keuls test. All statistical analyses were performed via SPSS 21.0 (IBM Co., Armonk, NY, USA). Differences of *P* < 0.05 (*) were considered statistical significant. *P* < 0.01 (# or **) and *P* < 0.001 (***) were considered statistically very significant.

## Results

### FC improves liver steatosis in HFD-induced obese mice

**Fig. 3:**
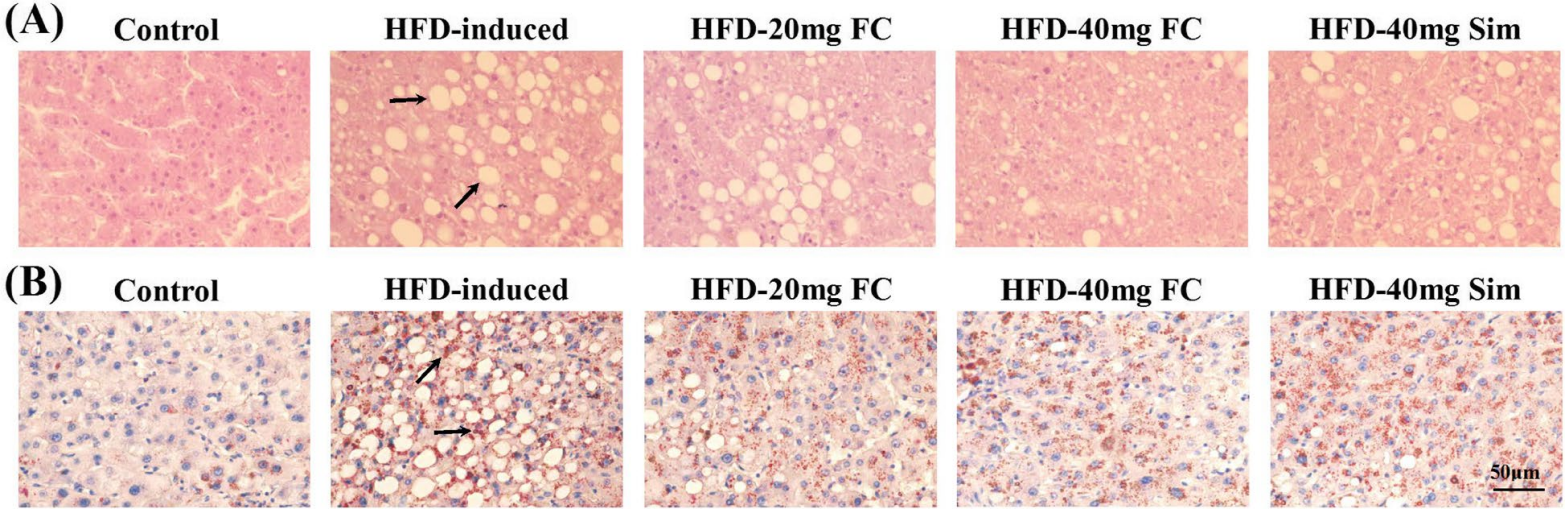
FC ameliorates the hepatic steatosis in HFD-induced obese mice. Histopathological analysis was exhibited via hematoxylin and eosin (H&E) staining **A**, and Oil-Red Stating **B**. HFD-induced liver steatosis obese mice showed abundant vacuoles lipid droplets in H & E staining (**A**, arrows) and oil red positive area in Oil-Red Staining (**B**, arrows). Simvastatin (Sim, 40 mg/kg) acted as the positive control group

Five-week-old C57BL/6 mice were fed with HFD diet for ten weeks the lipid data like the body weight (Fig. 2A), the liver weight (Fig. 2B), the liver/body weight ratio (Fig. 2C), hepatic TC (Fig. 2D), hepatic TG (Fig. 2E), and hepatic LDL-c (Fig. 2F) were significantly elevated. Thereafter the mice were treated with FC via intraperitoneal injection three times per week for ten weeks. As shown in Fig. 2A, significant differences in body weight and body weight gain were found between the control group and HFD-induced group (*P* < 0.01). Moreover, treatment with FC significantly reduced the body weight gain of the HFD-induced groups (Fig. 2B) (*P* < 0.05). The liver /body weight ratios of the HFD-induced group and the FC-treatment groups were significantly higher than that of the control group (Fig. 2C) (*P* < 0.05). The hepatic TC, TG, and LDL-c levels were also notably reduced in FC-treatment groups (Fig. 2D-F) (*P* < 0.05). The livers of HFD-induced mice revealed obvious lipid accumulation. Many vacuoles lipid droplets were examined by H & E staining (Fig. 3A, arrows). Great deals of oil red positive areas were detected by Oil-Red Staining (Fig. 3B, arrows). Nevertheless, FC could alleviate lipid deposition. Effect of HFD-20 mg FC could be comparable to positive reference HFD-40 mg Sim. These results demonstrated that FC improved liver steatosis in HFD-induced mice.

### FC ameliorates liver injury without significant adverse side effects

**Fig. 4:**
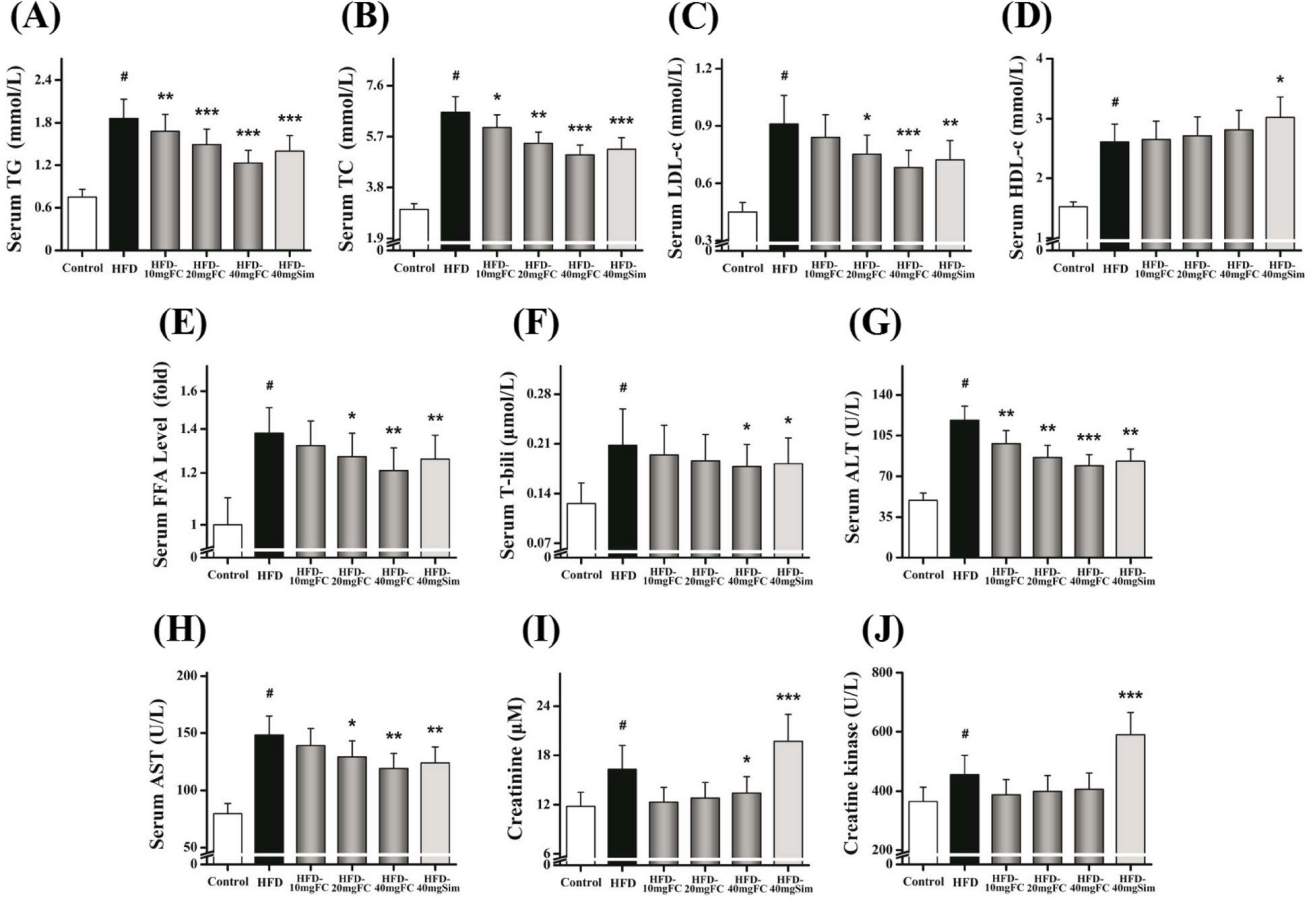
FC ameliorates liver injury without significant adverse side effects in HFD-induced obese mice. **A**-**E** The levels of serum TG **A**, TC **B**, LDL-c **C**, HDL-c **D**, and FFA **E** were measured. **F**–**H** Liver injury was evaluated via serum T-bili **F**, ALT **G**, and AST **H** levels. Adverse side effects of FC were examined via serum creatinine **I** and creatine kinase **J**. TG, Triglyceride; TC, total cholesterol; LDL-c, low density lipoprotein-cholesterol; HDL-c, high density lipoprotein-cholesterol; FFA, free fatty acid; T-bili, total bilirubin; ALT, alanine transaminase; AST, aspartate transaminase. Sim (40 mg/kg) acted as the positive control group. Each value represents as means ± S.E.M. of triplicate experiments. ^#^ *P* < 0.01 as compared with the control group. ^*^ *P* < 0.05, ^**^ *P* < 0.01, and ^***^ *P* < 0.001 as compared with the HFD-induced group

Since acute liver injury and myopathy were the most adverse side effects of statins administration reported previously [11, 12]. Thus, we measured some serum lipid profiles. As shown in Fig. 4A-E, the serum TG, TC, LDL-c, HDL-c, and FFA levels were all significantly elevated in HFD-induced group (*P* < 0.01). However, FC could dose-dependently attenuate these hepatic lipid metabolisms (*P* < 0.05). FC-treatment which reduced the levels of serum T-bili, ALT, and AST could markedly ameliorate liver injury (Fig. 4F-H) (*P* < 0.05). Meanwhile, prolonged statins administration had significant adverse side effects. The creatinine and creatine kinase levels were elevated in HFD-40 mg Sim group (*P* < 0.001). As shown in Fig. 4I-J, there was no difference of two parameters in FC-treatment groups. These data indicated that FC ameliorated liver injury without significant adverse side effects in HFD-induced mice.

### FC alleviates liver steatosis via attenuating de novo* lipogenesis*

**Fig. 5:**
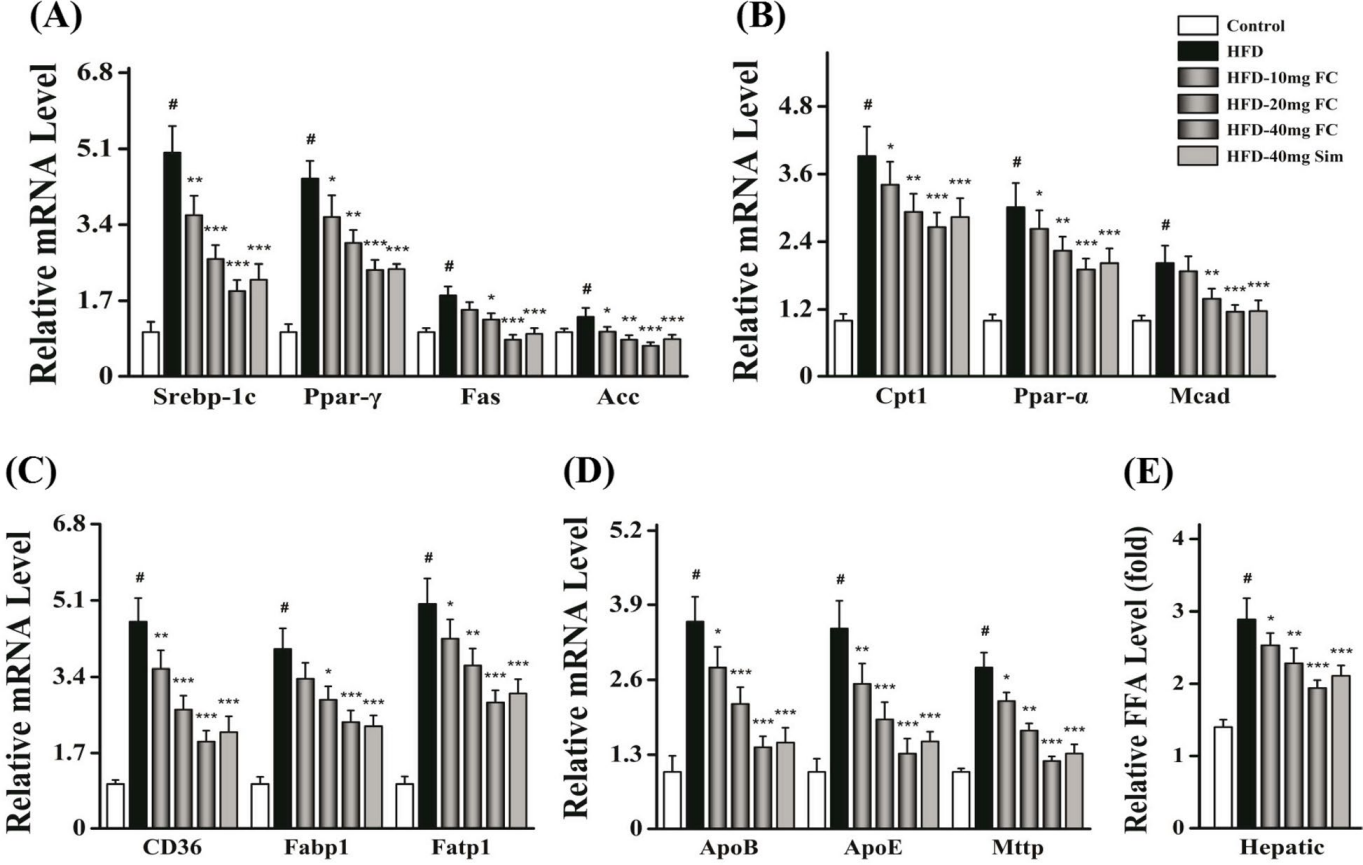
Effects of FC-treatment on the hepatic lipid metabolism-related genes expressions in HFD-induced obese mice. Hepatic lipid metabolism-related genes expressions, such as DNL **A**, β-oxidation **B**, FFA uptake **C** and VLDL export **D** were examined. **E** The level of hepatic FFA was measured. DNL, de novo* lipogenesis*; FFA, free fatty acid; VLDL, very low density lipoprotein; Srebp-1c, sterol response element binding protein-1c; Ppar, peroxisome proliferator activated receptor; Fas, fatty acid synthase; Acc, Acetyl-CoA; Cpt1, carnitine palmitoyltransferase 1; Mcad, medium chain acyl dehydrogenase; CD36, fatty acid translocase; Fabp1, fatty acid binding protein 1; Fatp1, fatty acid transport protein 1; ApoB, Apo lipoprotein B; ApoE, Apo lipoprotein E; Mttp, microsomal triglyceride transfer protein. Sim (40 mg/kg) acted as the positive control group. Each value represents as means ± S.E.M. of triplicate experiments. ^#^ *P* < 0.01 as compared with the control group. ^*^ *P* < 0.05, ^**^ *P* < 0.01, and ^***^ *P* < 0.001 as compared with the HFD-induced group

Substantial evidences have revealed that the level of hepatic TG depended on de novo* lipogenesis*, β-oxidation, FFA uptake, and very low density lipoprotein (VLDL) export [36, 37]. To evaluate which the most possible pathway involved in FC-treatment, we determined the hepatic lipid metabolism-related genes expressions involved in these four stages. Interestingly, FC could markedly and dose-dependently inhibit the de novo* lipogenesis* related mRNA expressions such as sterol response element binding protein-1c (Srebp-1c), peroxisome proliferator activated receptor-γ (Ppar-γ), fatty acid synthase (Fas), and Acetyl-CoA (Acc) (Fig. 5A) (*P* < 0.05). Furthermore, FC reduced the β-oxidation related mRNA expressions like carnitine palmitoyltransferase 1 (Cpt1), peroxisome proliferator activated receptor-α (Ppar-α), and medium chain acyl dehydrogenase (Mcad) (Fig. 5B) (*P* < 0.05). Moreover, FC attenuated the FFA uptake related mRNA expressions including fatty acid translocase (Fat/CD36), fatty acid binding protein 1 (Fabp1), and fatty acid transport protein 1 (Fatp1) (Fig. 5C) (*P* < 0.05). In addition, FC decreased the VLDL related mRNA expressions like Apo lipoprotein B (ApoB), Apo lipoprotein E (ApoE), and microsomal triglyceride transfer protein (Mttp) (Fig. 5D) (*P* < 0.05). As shown in Fig. 5E, FC-treatment could reverse gradually hepatic FFA content of HFD-induced mice (*P* < 0.05). These results concluded that FC could dose-dependently modulate the expressions of lipid metabolism-related transcription genes via attenuating hepatic de novo* lipogenesis* and FFA uptake.

### FC inhibits Srebp-1c mediated de novo* lipogenesis* through modulating of the RhoA/ROCK signaling pathway

**Fig. 6:**
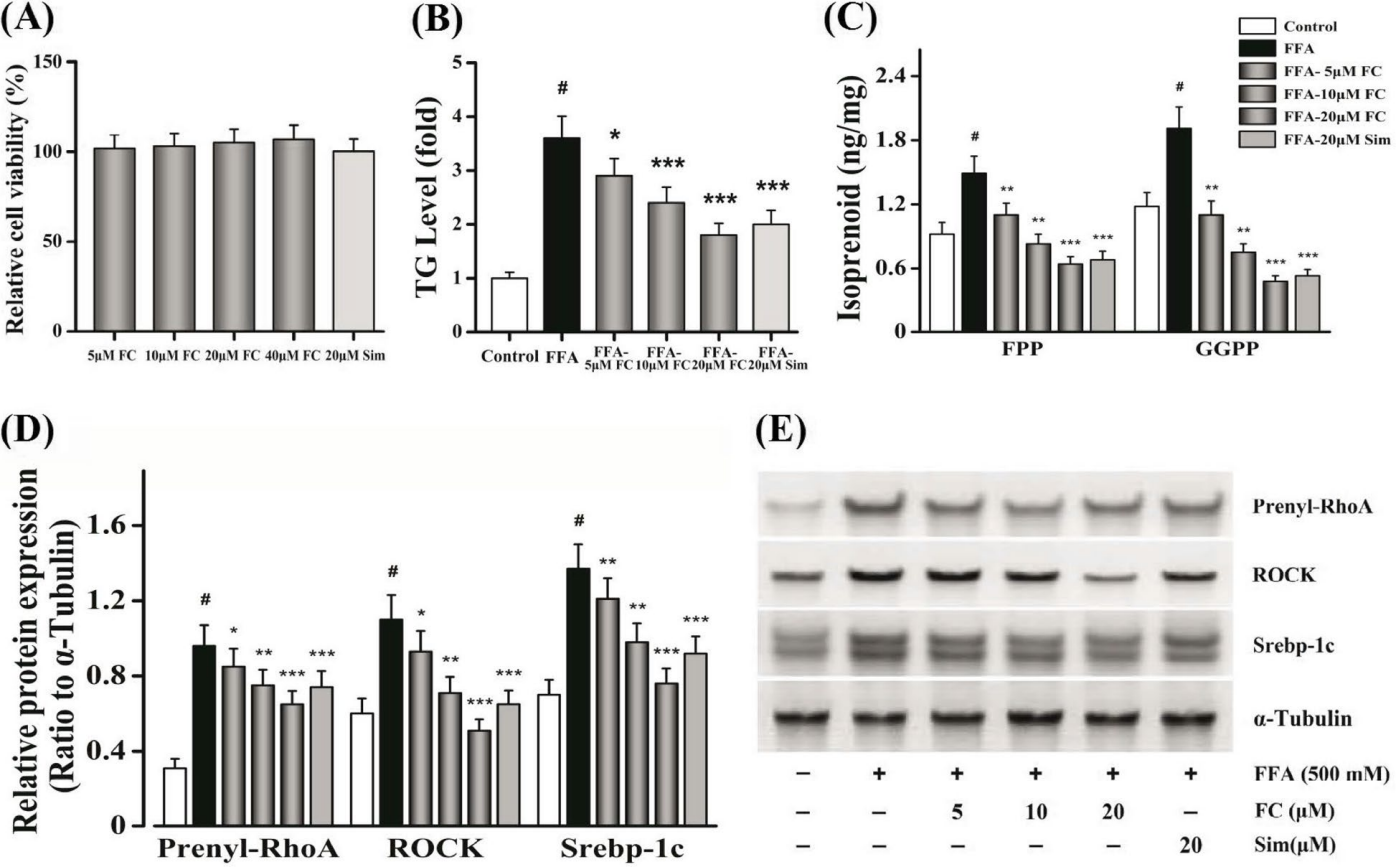
FC suppresses Srebp-1c mediated hepatic DNL by modulating the RhoA/ROCK signaling pathway in FFA-induced AML-12 cell. **A** FC-treatment on AML-12 cell viability. **B** The level of hepatocellular TG was examined. **C** The levels of FPP and GGPP were measured by HPLC–MS/MS. **D**, **E** Western blotting technique was used to evaluate the expressions of prenyl-RhoA, ROCK, and Srebp-1c. FFA, free fatty acid; TG, Triglyceride; FPP, farnesyl diphosphonate; GGPP, geranylgeranyl diphosphonate; Srebp-1c, sterol response element binding protein-1c. Sim (20 μM) acted as the positive control group. Each value represents as means ± S.E.M. of triplicate experiments. ^#^ *P* < 0.01 as compared with the control group. ^*^ *P* < 0.05, ^**^ *P* < 0.01, and ^***^ *P* < 0.001 as compared with the FFA-induced group

Some previous researches have reported that de novo* lipogenesis* was controlled via activating the expressions of Srebp-1c [38, 39]. To investigate the mechanism of which FC could modulate Srebp-1c-related pathways, we further evaluated Srebp-1c-related kinases. As shown in Fig. 6A, the tested concentrations of FC showed no obvious cytotoxicity in AML-12 cells. FC could remarkably and dose-dependently reduce the TG level in AML-12 cells (Fig. 6B) (*P* < 0.05). Moreover, the levels of GGPP and FPP were decreased after FC-treatment in AML-12 cells (Fig. 6C) (*P* < 0.01). Furthermore, FC notably suppressed the maturation of Srebp-1c. The RhoA prenylation and the expression of ROCK, which is a downstream effector of RhoA, were also attenuated by FC exposure (Fig. 6D-E) (*P* < 0.05). Together these data suggested that FC inhibited Srebp-1c mediated de novo* lipogenesis* via interfering with the RhoA/ROCK signaling pathway by decreasing the levels of GGPP and FPP.

## Discussion

Currently, various alternative NAFLD therapeutic strategies including lifestyle intervention and pharmaceutical treatment are either insufficient or have side effects [5, 6]. Emerging researches have focused on natural products as a selectable effective therapeutic strategy without significant adverse side effects [18, 19]. In the present study, we investigated the potential NAFLD therapeutic effect of FC. Our data demonstrated that FC could ameliorate hepatic steatosis and alter GGPP and FPP levels both in vitro and in vivo without obvious adverse side effects. The mechanisms were involved in inhibiting de novo* lipogenesis*, altering the levels of GGPP and FPP, and modulating the RhoA/ROCK signaling pathway.

**Fig. 7:**
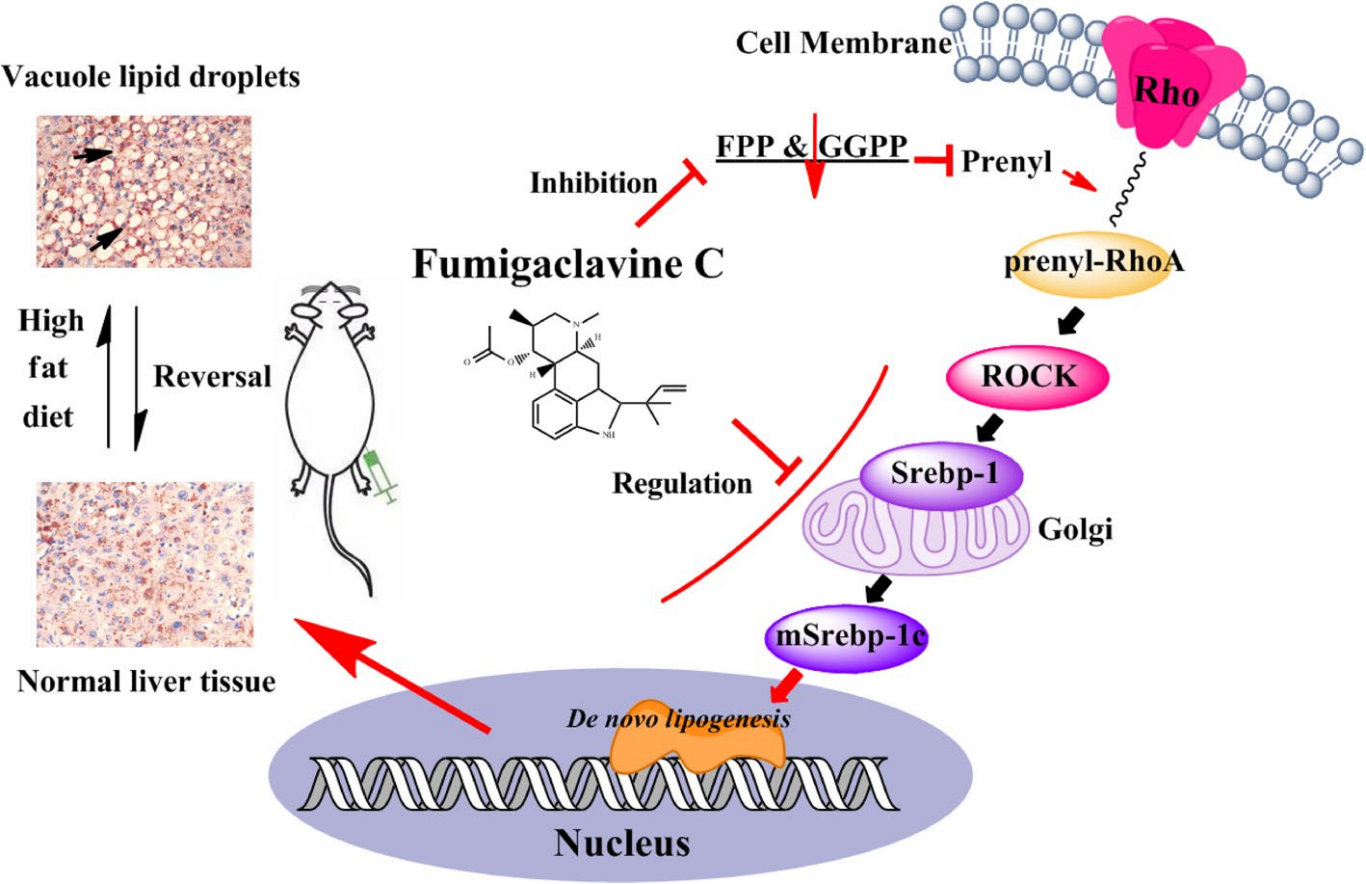
Scheme of the molecular mechanisms of the hepatoprotective effects of FC

Substantial evidences have revealed that the level of hepatic TG depended on de novo* lipogenesis*, β-oxidation, FFA uptake, and VLDL export [36, 37]. Thus, we tried to elucidate which the possible pathway FC took part in attenuating hepatic lipid accumulation. First of all, FC could markedly and dose-dependently inhibit the mRNA expressions of de novo* lipogenesis*, particularly in HFD-40 mg FC group. As FC-treatment notably suppressed the levels of hepatic TG and FFA, it is reasonable to unravel that the hepatic TG reduction cause by attenuating de novo* lipogenesis.* Secondly, de novo* lipogenesis* contributed to twenty-six percent of TG deposition, FFA uptake accounted for fifty-nine percent in NAFLD [36]. Some studies have reported that normal subjects were one-third of rates of de novo fatty acid synthesis compared with NAFLD subjects [37]. The livers of HFD-induced mice revealed obvious lipid accumulation [40]. Nevertheless, FC could alleviate lipid deposition [41, 42]. Hence, we next assessed whether FC reduced hepatic lipid accumulation via interfering with de novo* lipogenesis*. Thirdly, some previous researches have reported that de novo* lipogenesis* was controlled via activating the expressions of Srebp-1c [38, 39]. To investigate the mechanism of which FC could modulate Srebp-1c-related pathways, we further evaluated Srebp-1c-related kinases. Srebp-1c was suggested to be cleaved to be maturated under the RhoA/ROCK signaling pathway [13]. Especially, Rho need to undergo prenylation to be activated [14]. In this study, the levels of GGPP and FPP were decreased after FC-treatment in AML-12 cells. This might be due to the reduction of the maturation of Srebp-1c via blocking the RhoA prenylation and the expression of ROCK (Fig. 7). Together these data suggested that FC inhibited Srebp-1c mediated de novo* lipogenesis* via interfering with the RhoA/ROCK signaling pathway by decreasing the levels of GGPP and FPP.

Until now, great administrations for NAFLD have been always limited [16, 17]. Statins, HMG-CoA reductase inhibitor, had been usually used for management of NAFLD-associated hypercholesterolemia [9, 10]. However, long-term statins treatment had side effects including hepatotoxicity and myopathy [11, 12], due to depletion of cholesterol, GGPP, and FPP at the beginning of mevalonate pathway [13, 14]. In this study, some results demonstrated that FC could not only ameliorate liver steatosis, but also do not worsen, as well as improve obesity-induced liver injury in HFD -induced obese mice. Possible adverse side effects of FC were not reported, such as myalgia, fatigue, hepatotoxicity, and fever [21–30]. Interestingly, FC has scarcely obvious adverse side effects. There was no difference in the levels of serum ALT, AST, T-bili, creatinine, and creatine kinase in FC-treatment groups. These data indicated that FC ameliorated liver injury without significant adverse side effects in HFD-induced mice.

Furthermore, there are still many limitations in this study. Besides RhoA-prenylation, other small G proteins (such as Rab and Rac1) and G-γ subunit of GTPase proteins also need to go through prenylation and subsequent membrane-association to be activated [13–15]. Hence, it is very important to further research the potentiality of FC on other G-γ subunit of GTPase proteins and small G proteins. This research only provides a potential way to ameliorate liver steatosis with FC-treatment, while the precise molecular mechanisms remain to be elucidated.

## Conclusion

This study elucidated that FC could ameliorate hepatic steatosis and alter GGPP and FPP levels both in vitro and in vivo without obvious adverse side effects (Fig. 7). The mechanisms were involved in inhibiting de novo* lipogenesis*, altering the levels of GGPP and FPP, and modulating the RhoA/ROCK signaling pathway.

### Supplementary Information

**Additional file 1: Supplementary Information 1.**

**Additional file 2: Supplementary Information 2.**

## Data Availability

The datasets used and/or analyzed during the current study are available from the corresponding author on reasonable request.

## Abbreviations

NAFLD: Non-alcoholic fatty liver disease
NASH: Nonalcoholic steatohepatitis
FC: Fumigaclavine C
HFD: High-fat diet
RC: Regular chow
GGPP: Geranylgeranyl diphosphate
FPP: Farnesyl diphosphate
Sim: Simvastatin
Srebp-1c: Sterol response element binding protein-1c
TC: Total cholesterol
TG: Triglyceride
LDL-c: Low density lipoprotein-cholesterol
HDL-c: High density lipoprotein-cholesterol
FFA: Free fatty acid
T-bili: Total bilirubin
ALT: Alanine transaminase
AST: Aspartate transaminase
CCK-8: Cell Counting Kit-8
DMSO: Dimethyl Sulphoxide
H & E: Hematoxylin & eosin
PVDF: Polyvinylidene difluoride
DNL: De novo* lipogenesis*
VLDL: Very low density lipoprotein
Ppar: Peroxisome proliferator activated receptor
Fas: Fatty acid synthase
Acc: Acetyl-CoA
Cpt1: Carnitine palmitoyltransferase 1
Mcad: Medium chain acyl dehydrogenase
CD36: Fatty acid translocase
Fabp1: Fatty acid binding protein 1
Fatp1: Fatty acid transport protein 1
ApoB: Apo lipoprotein B
ApoE: Apo lipoprotein E
Mttp: Microsomal triglyceride transfer protein
