# Supplementary figures and images for: Fumigaclavine C ameliorates liver steatosis by attenuating hepatic de novo lipogenesis via modulation of the RhoA/ROCK signaling pathway
Source: BMC Complement Med Ther. 2023 Aug 16;23:288. doi: 10.1186/s12906-023-04110-9 (PMC10428638; doi:10.1186/s12906-023-04110-9)

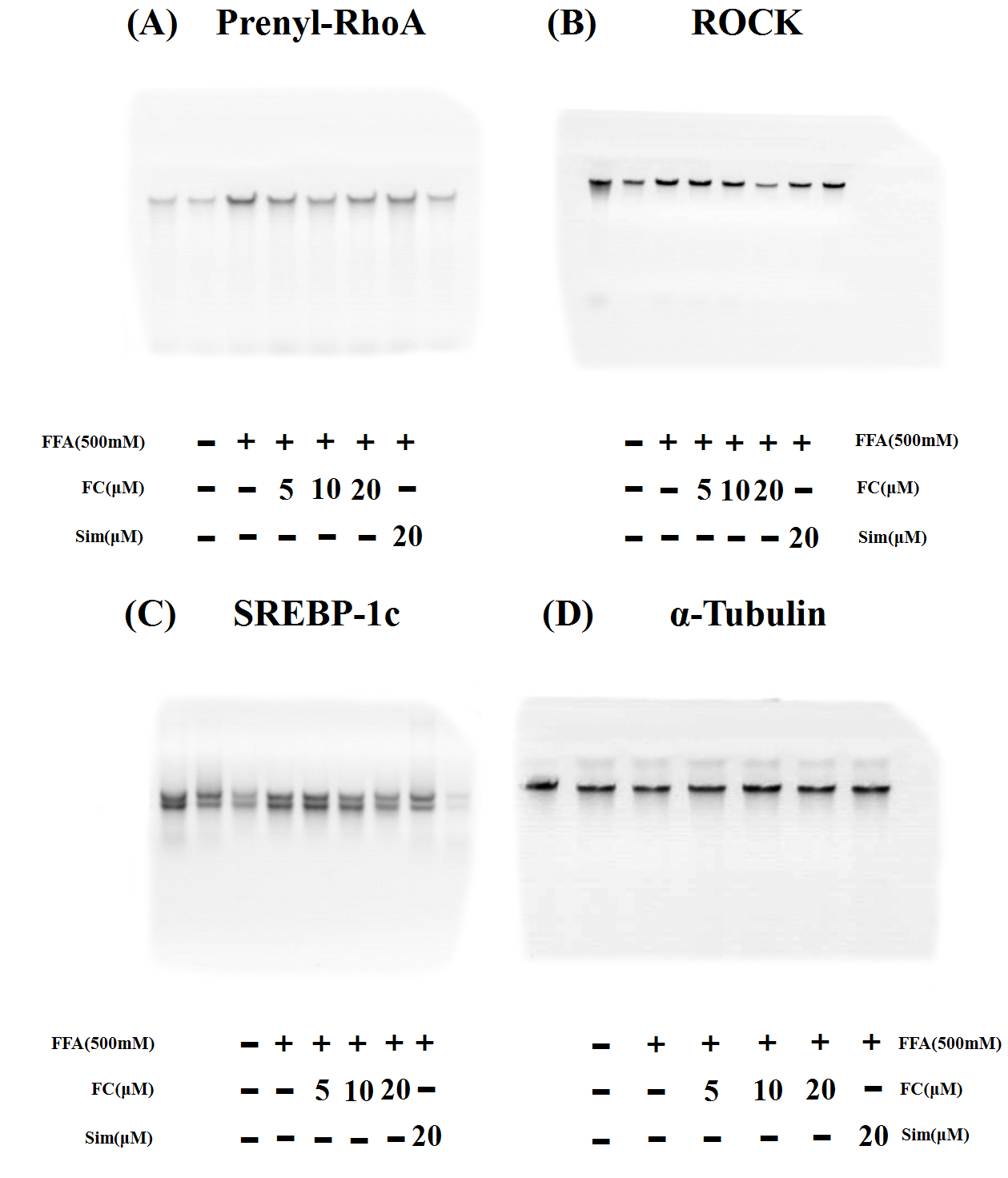

Supplement: Supplementary file 1 — Additional file 1: Supplementary Information 1. [file 12906_2023_4110_MOESM1_ESM.tif]
