# Supplementary material for: Fumigaclavine C ameliorates liver steatosis by attenuating hepatic de novo lipogenesis via modulation of the RhoA/ROCK signaling pathway
Source: BMC Complement Med Ther. 2023 Aug 16;23:288. doi: 10.1186/s12906-023-04110-9 (PMC10428638; doi:10.1186/s12906-023-04110-9)
Supplement: Supplementary file 2 — Additional file 2: Supplementary Information 2. [file 12906_2023_4110_MOESM2_ESM.docx]

**Supplementary Information**

Supplementary Information 1 is Original and unprocessed version of Western blotting.

**Abbreviations**

FFA Free fatty acid

FC Fumigaclavine C

Sim Simvastatin

Table 1. The levels of serum TG, TC, LDL-c, HDL-c, FFA, T-bili, ALT, AST, creatinine, and creatine kinase levels.

|  | Normal reference range values | Baseline values |
| --- | --- | --- |
| TG (mmol/L) | 0.637 ~ 0.863 | 0.750 |
| TC (mmol/L) | 2.533 ~ 3.427 | 2.980 |
| LDL-c (mmol/L) | 0.383 ~ 0.518 | 0.451 |
| HDL-c (mmol/L) | 1.292 ~ 1.748 | 1.520 |
| FFA (fold) | 0.835 ~ 1.125 | 0.980 |
| T-bili ((μmol/L)) | 0.107 ~ 0.145 | 0.126 |
| ALT (U/L) | 42.15 ~ 56.92 | 49.52 |
| AST (U/L) | 67.12 ~ 90.80 | 78.96 |
| Creatinine (μM) | 10.05 ~ 13.55 | 11.80 |
| Creatine kinase (U/L) | 311.3 ~ 421.1 | 366.2 |

TG, Triglyceride; TC, total cholesterol; LDL-c, low density lipoprotein-cholesterol; HDL-c, high density lipoprotein-cholesterol; FFA, free fatty acid; T-bili, total bilirubin; ALT, alanine transaminase; AST, aspartate transaminase.
